# Sea Sickness

**Published:** 1896-12

**Authors:** F. H. Lutze

**Affiliations:** Cheshire, N. Y.


### SEA SICKNESS.

CHESHIRE, N. Y., November 2d, 1896.

EDITOR OF THE HOMŒOPATHIC PHYSICIAN:

In the May number of your journal, p. 243, I noticed Dr. H. N. Guernsey's "Therapeutic Indications for Sea Sickness."

I send you enclosed an article to supplement that of Dr. Guerney for publication. I had opportunities on a trip across the Atlantic to test the value of *Ars.*, *Borax*, *Calc-c.*, *Nux-v.*, *Sepia*, and can vouch for their prompt and efficient action.

Fraternally,

F. H. LUTZE.

---

### SEA SICKNESS.

F. H. LUTZE, M. D., CHESHIRE, N. Y.

*Arsenic.*—Thirst for *cold water*, but vomits immediately after drinking. The smell and sight of food cause or increase the nausea. Desire for warmth, warm open air on deck; restlessness, etc.

*Borax.*—*Aggravation of downward motion.* Every time the vessel goes downward, everything within me wants to come up. (Two powders of the Borax$^{200}$ cured, after patient had been sick two-thirds of the voyage, and the rough weather still continuing.)

*Calcarea-ost.*—*Aggravation from upward* motion; sour water flows from the mouth, with nausea, or collects in the mouth; great thirst for cold drinks, especially water. No appetite; milk or meat do not taste good. Nausea in the pit of the stomach from a sensation of emptiness. Vertigo.

*Cocculus.*—The nausea is felt mostly in the head, and the stomach seems to heave up and down; nausea from looking at the pitching of the vessel and a tendency to faint; (nausea from riding in a car or carriage, especially when riding backward).

*Colchicum.*—Nausea and vomiting from the smell of the cooking, feels much better from lying perfectly quiet.

*Nux-vom.*—Death-like pallor of the face, ineffectual urging to vomit or to stool; thinks he would feel so much better if he could only vomit. Worse mornings.

*Sepia.*—Much nausea and vomiting, especially mornings before breakfast and forenoon; aversion to the smell of cooking and even the sight of food; (nausea from riding in a carriage). Sensation as if the contents of the abdomen were turning over and over. Desire for sour, refreshing things; nausea from rinsing out the mouth; water collects in the mouth; sour, bitter taste, better from breakfast. Food tastes natural; costive.

*Petroleum.*—Great sensation of emptiness in the stomach; nausea early in the morning, with collection of water in the mouth; vertigo, with heat in the face; nausea and vertigo all day; desire for beer.

*Pulsatilla.*—Chilly, dizzy, and sleepy; no thirst; vertigo on rising from a seat; all better by remaining up on deck in the open air.

*Opium.*—Great sleepiness and constipation; absence of any desire, or ineffectual desire, for stool.

